# A high-throughput 3D cantilever array to model airway smooth muscle hypercontractility in asthma

**DOI:** 10.1063/5.0132516

**Published:** 2023-05-16

**Authors:** Pranjali Beri, Christopher Plunkett, Joshua Barbara, Chien-Cheng Shih, S. Whitney Barnes, Olivia Ross, Paula Choconta, Ton Trinh, Datzael Gomez, Bella Litvin, John Walker, Minhua Qiu, Scott Hammack, Erin Quan Toyama

**Affiliations:** Novartis Institutes for Biomedical Research, San Diego, California 92121, USA

## Abstract

Asthma is often characterized by tissue-level mechanical phenotypes that include remodeling of the airway and an increase in airway tightening, driven by the underlying smooth muscle. Existing therapies only provide symptom relief and do not improve the baseline narrowing of the airway or halt progression of the disease. To investigate such targeted therapeutics, there is a need for models that can recapitulate the 3D environment present in this tissue, provide phenotypic readouts of contractility, and be easily integrated into existing assay plate designs and laboratory automation used in drug discovery campaigns. To address this, we have developed DEFLCT, a high-throughput plate insert that can be paired with standard labware to easily generate high quantities of microscale tissues *in vitro* for screening applications. Using this platform, we exposed primary human airway smooth muscle cell-derived microtissues to a panel of six inflammatory cytokines present in the asthmatic niche, identifying TGF-β1 and IL-13 as inducers of a hypercontractile phenotype. RNAseq analysis further demonstrated enrichment of contractile and remodeling-relevant pathways in TGF-β1 and IL-13 treated tissues as well as pathways generally associated with asthma. Screening of 78 kinase inhibitors on TGF-β1 treated tissues suggests that inhibition of protein kinase C and mTOR/Akt signaling can prevent this hypercontractile phenotype from emerging, while direct inhibition of myosin light chain kinase does not. Taken together, these data establish a disease-relevant 3D tissue model for the asthmatic airway, which combines niche specific inflammatory cues and complex mechanical readouts that can be utilized in drug discovery efforts.

## INTRODUCTION

Chronic asthma is characterized by permanent structural changes to the airways known as airway remodeling, which consists of modifications to epithelial cells, smooth muscle, and fibroblasts, as well as changes in the extracellular matrix (ECM) composition.^1^ This remodeling coincides with an elevated presence of immune cells within the asthmatic niche, including T-Helper lymphocytes, eosinophils, mast cells, and macrophages, which, in turn, leads to increased levels of inflammatory cytokines.^2^ Signaling from these cytokines has been associated with dysfunction in several airway cell types, including apoptosis and EMT in the mucosal epithelium and subepithelial fibrosis emerging from activated lung fibroblasts.^3^ More recently, the focus of therapeutic drug discovery in asthma has shifted toward the role of the airway smooth muscle,^4^ whose structural changes have been linked to hyperplasia, hypertrophy, and increases in bronchoconstriction.^1,5^ The latter consists of both a baseline increase in airway tightness and narrowing due to smooth muscle hypercontractility and remodeling,^5–7^ as well as increased sensitivity in response to triggers of bronchoconstriction.^5^ The airways of asthmatic subjects have been shown to have a higher airway resistance at maximal inspiration compared to non-asthmatics, likely due to the increased contractile state of the airway smooth muscle.^8^ In cases of severe asthma (5%–10% of the patient population) with poor response to inhaled corticosteroids or bronchodilators, such as LABA/LAMAs (long-acting beta-agonist/long-acting muscarinic antagonist),^9,10^ bronchial thermoplasty, or the removal of excess smooth muscle to improve baseline relaxation of the airway, is an option, but this procedure is risky and might not provide long-term relief.^10^ Therefore, it is essential to identify therapies to reduce the remodeling induced by hypercontractile airway smooth muscle present in asthma.

Contractility in airway smooth muscle is largely governed by the dynamics of actin and myosin filaments within the cytoskeleton.^11^ Phosphorylation of myosin's light chain (MLC) region enables its molecular interaction with actin, resulting in force generation within the cell. Phosphorylation and dephosphorylation of MLC is catalyzed by myosin light chain kinase (MLCK) and myosin light chain phosphatase (MLCP), respectively, and it is their interplay that governs contracted or relaxed cell states.^12^ MLCK activity is believed to be largely driven by extracellular and intracellular calcium release,^13,14^ while MLCP regulation is associated with several upstream signaling pathways, including its well-established inhibition by rho-associated protein kinase (ROCK) and inhibition by protein kinase C (PKC) via phosphorylation of the protein CPI-17.^15,16^ However, despite these well-defined mechanisms, the role of inflammatory signaling in the asthmatic niche toward modulating smooth muscle contractility remains unclear.

To address this, complex *in vitro* modeling can allow for an assay design that replicates the core phenotypic readouts found *in vivo*, such as BSMC contractile force generation, and allows for systematic testing of notable inflammatory signals found in the niche. Previous *in vitro* BSMC studies have utilized single-cell contraction assays, which demonstrate that cells derived from asthmatic patients are more contractile than those from healthy patients^17,18^ and exhibit greater contraction in response to external stimuli such as histamine.^19^ Gel contraction assays have also been used to show that co-culture of airway smooth muscle cells with mast cells increases gel contraction and is dependent on matrix remodeling, suggesting a link between inflammatory mediators, contractility, and airway remodeling.^20^ However, a major challenge in drug discovery when screening for complex mechanical phenotypes, such as hypercontractility, is the lack of high-throughput systems that can both simulate the desired mechanics *in vitr*o and be easily integrated with existing automation systems and high-content imagers.^7,21^ While there are several hydrogel-based *in vitro* models for modeling contractility, such as simple gel contraction assays^22^ and traction force microscopy,^23^ miniaturization is limited by design challenges such as poor attachment of the gel to the well bottom, interference of gel meniscus with imaging, complex analysis protocols, and low signal to noise ratios. There is also difficulty in scaling down to high-throughput arrays due to poor integration with existing high-content imagers and liquid handlers. Additionally, the desire for more physiologically relevant phenotypic screening has led to the incorporation of complex 3D structures with human cells to increase the translatability of preclinical data and accelerate drug discovery.^24,25^

Elastomer-based cantilever strain gauges have emerged as a powerful means to model gross multi-cellular contractile forces in 3D at both the macro and microscales.^26^ An improvement in the design of these tools is the inverted “hanging” style of construction whereby tissues are suspended from cantilevers that are mounted to the top of the cell culture multi-well plate, which greatly simplifies tissue casting and improves imaging resolution.^27,28^ These bioengineered tissue systems, however, are often lower in total throughput than highly multiplexed experiments because they require and utilize formats incompatible with existing microwell plate-based laboratory robotics and instrumentation.^29^ The result is a lack of available 3D systems that can be easily integrated into existing screening protocols, which, in turn, limits the scope of drug discovery-focused experimental designs and broad platform adoption for target ID studies.

To address these challenges, we developed a 96-well device enabling functional linear contractility of tissues (DEFLCT) for multiplexed disease modeling of microtissue structures comprised of contractile cells. This system is simple to construct in high volumes, is fully compatible with off the shelf assay plates, and leverages hanging construction for reproducible tissue casting. We then utilized this platform to expose airway smooth muscle cell microtissues to a panel of inflammatory cytokines to induce smooth muscle shortening via increased contractility. Finally, we validated the shift toward an asthmatic disease phenotype through RNAseq, which revealed enrichment of pathways in the remodeled tissues that are associated with asthma, airway remodeling, and contractility.

## RESULTS

### Development of a 96-well ANSI/SLAS compatible cantilever system

**FIG. 1.**
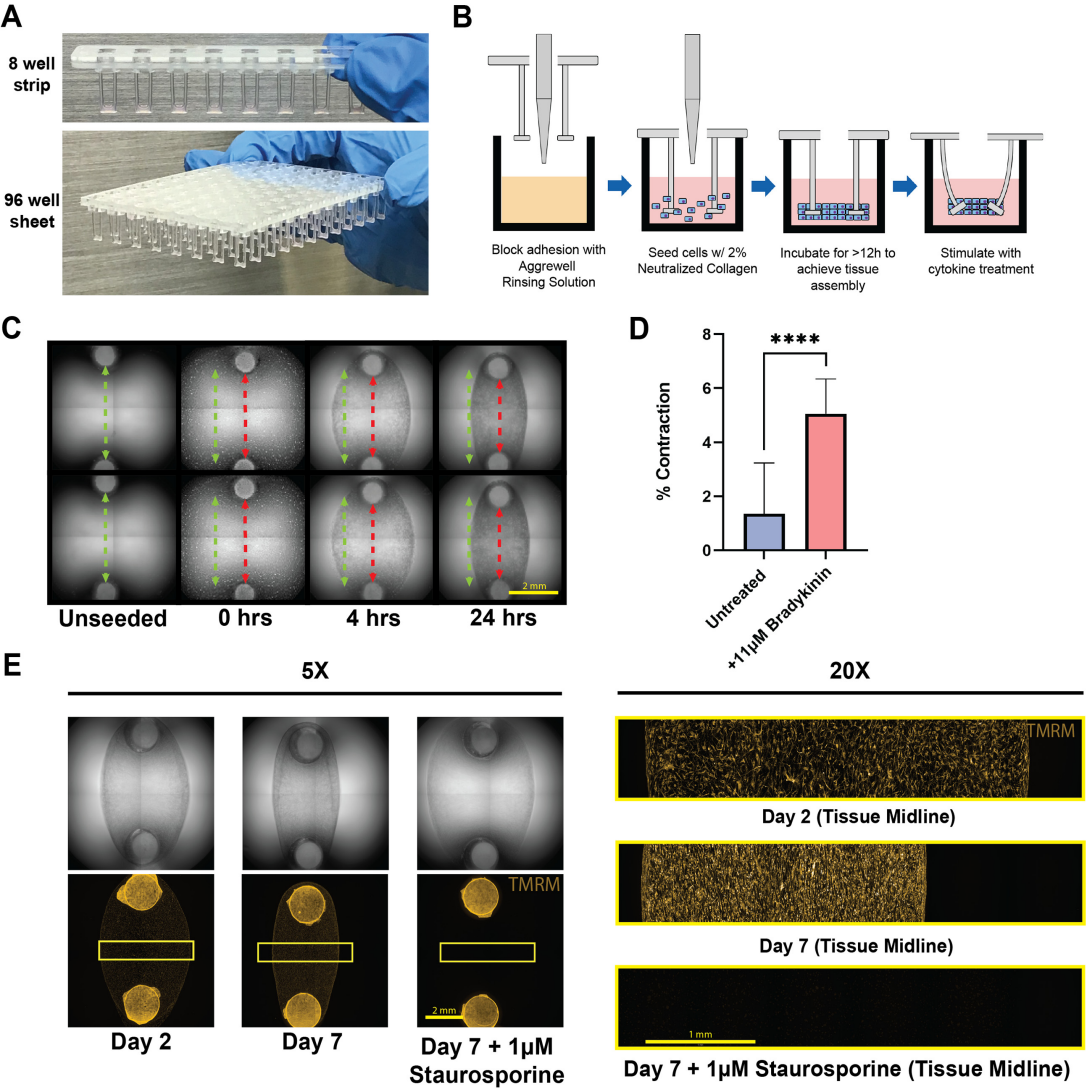
Overview of DEFLCT system design and general performance. (a) Fully assembled and seeded DEFLCT consumables for 8- and 96-well formats. Parts are constructed from a silicone elastomer pillar array coupled to a rigid acrylic backing, allowing placement inside standard ANSI/SLAS compliant assay plates. Individual 8-well strips are utilized for limited throughput studies, while full 96-well arrays are intended for screening experiments. (b) Process overview for seeding DEFLCT systems. Standard 96-well labware is coated with an adhesion blocking buffer after which cells are added to the well along with a pre-polymerized hydrogel. The gel is then allowed to cure around the posts forming the tissue and causes baseline deflection of the cantilevers within 24 h. (c) Timelapse image of tissue formation and tightening on cantilevers over a 24 h period. Initial cantilever spacing (green) is reduced over the formation period during consolidation of the hydrogel and tightening of the seeded cells (red). Scale bar = 2 mm. (d) Tissue contraction percentage after treatment with 100 μM bradykinin following a 12 min incubation period. Data represents two biological replicates comprised of at least eight technical replicates each. Bars represented as mean ± S.E.M. and analyzed via unpaired t-test with Welch's correction. ^****^p < 0.0001. (e) TMRM live cell staining of tissues cultured at days 2 and 7 relative to dead control (1 *μ*M staurosporine). 5× scale bar = 2 mm and 20× scale bar = 1 mm.

To achieve sufficient assay throughput for parallelized multi-stimuli screening of contractility, we developed a 96-well format cantilever array to culture airway smooth muscle 3D microtissues. The device functions as a plate insert hanging down into the individual wells, which allows for the use of standard ANSI/SLAS (Society of Biomolecular Screening) dimensioned labware and existing high content laboratory instruments [Fig. 1(a) and supplementary material Fig. 1(a)]. To achieve this high density of microscale features while maintaining physiologically relevant rigidity, we elected to utilize NuSil MED-4940 silicone resin, which has an equivalent stiffness to the more commonly utilized Dow Corning Sylgard 184 but maintains a threefold higher ultimate tensile strain, allowing for aggressive demolding without feature damage [supplementary material Figs. 1(b)–1(d)]. Due to the significantly higher viscosity of MED-4940 precursor resin, pressurized molding into an aluminum form was employed to fabricate consumables [supplementary material Fig. 2(a)]. Computational modeling of this cantilever design yielded an estimated spring constant of 0.109 *μ*N/*μ*m and demonstrated symmetrical, linear deflections in response to applied force [supplementary material Figs. 2(b) and 2(c)].

Tissue casting on DEFLCT followed a straightforward four step process, where following adhesion blocking of the assay plate, a mixture of cells and neutralized collagen was added to the wells. Then, the DEFLCT consumable was inserted into the plate and the mixture was allowed to polymerize for 15 min at 37 °C before adding growth media and incubating overnight [Fig. 1(b)]. Multiple seeding conditions were tested to optimize microtissue formation, with the tightest tissues derived from 50 000 cells suspended in a 1 mg/ml collagen gel solution (supplementary material Fig. 3). Given these conditions, tissues began compacting within 4 h of initial seeding and had fully consolidated by 24 h [Fig. 1(c)]. Applied across a full assay plate, this casting method was successfully achievable for tissue formation on all 96 cantilever pairs across multiple patient-derived doner cell lines [supplementary material Fig. 4(a)]. Similar to analogous *in vitro* contraction assay platforms for bSMCs,^17^ acute tissue contractions could be observed following treatment with 11 *μ*M bradykinin and a subsequent 12 min incubation period, demonstrating sensor responsiveness to externally applied contractile stimuli [Fig. 1(d)]. Tissues cultured on the consumable remained viable up to our maximum experimental time course of 7 days after initial seeding with live cells distributed across the tissue's cross section and thickness [Fig. 1(e) and supplementary material Fig. 4(b)].

### TGF-***β***1 and IL-13 induce contractile phenotypes in patient-derived hBSMCs

The compatibility of this system with existing well-plates and the 96-well format enables high-throughput, parallelized investigation of the effects of chemical stimuli (compounds, chemokines, etc.) on 3D microtissues of contractile cells to generate disease models and investigate potential therapies for diseases involving contractile cells. To investigate smooth muscle contractility in asthma, we applied these high-throughput capabilities to study changes in the contractile phenotype of primary human bronchial smooth muscle cells after treatment with TGF-β1,^30^ IL-13,^31^ TNFα,^32^ IL-1β,^33^ IL-17A,^21^ and INFγ,^34^ a panel of six inflammatory cytokines associated with airway inflammation in asthma and linked to airway smooth muscle contractility in previous literature.

**FIG. 2.**
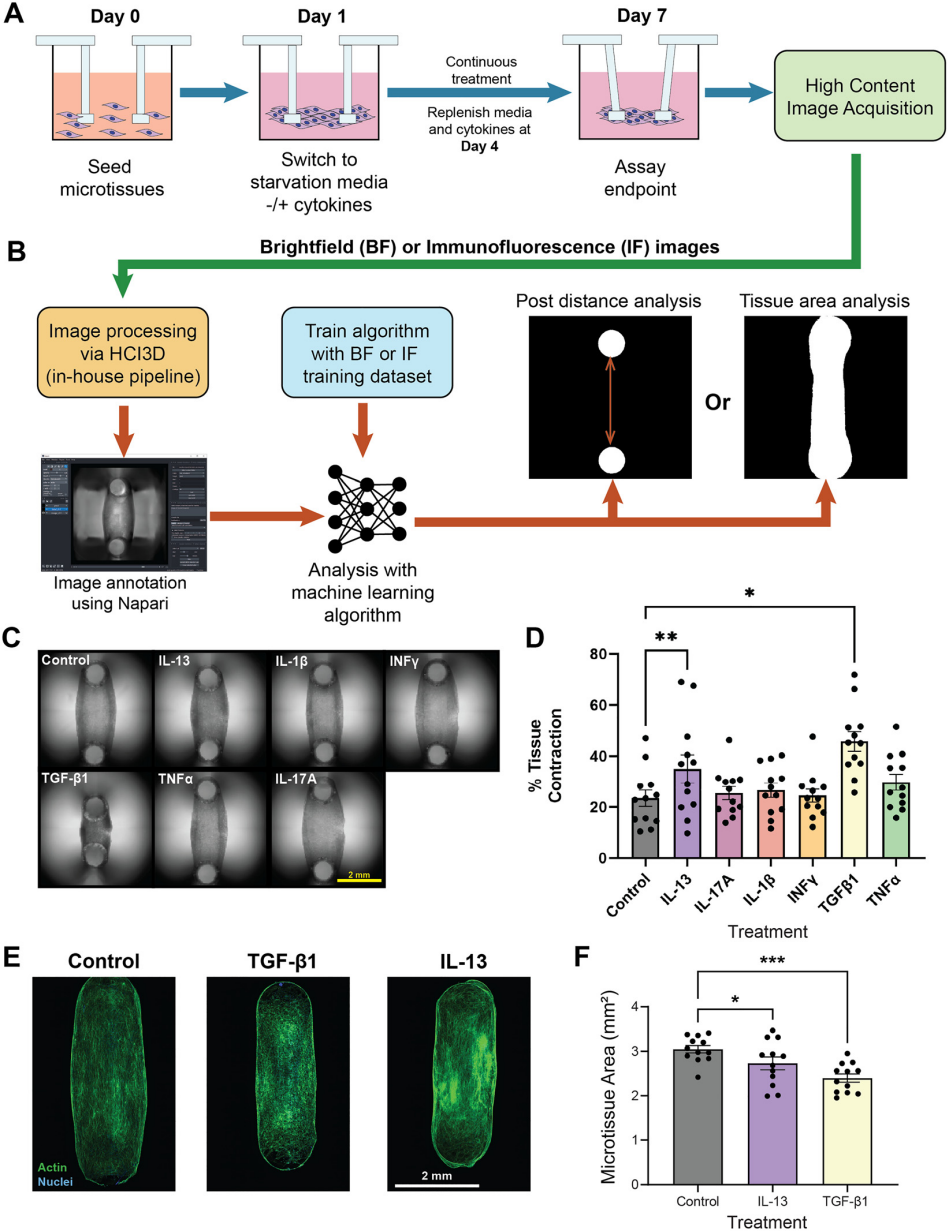
TGF-β1 and IL-13 induce contractile phenotypes in bSMC microtissues cultured on DEFLCT arrays. (a) Graphical abstract of experimental procedure and key assay timepoints. (b) Overview of machine vision image processing approach for contraction and tissue area readout. (c) Representative brightfield images of tissues treated with experimental cytokines at day 7. Scale bar = 2 mm. (d) Percentage (%) of microtissue contraction relative to initial pillar spacing for cytokine treatment conditions. All individual data points represent biological replicates that were averaged from ≥ 3 technical replicates each. Conditions are comprised of three biological replicates for four human donors (n = 12 microtissues per condition). Data represented as mean ± S.E.M. and analyzed by one way ANOVA with Geisser–Greenhouse correction and Dunnett's multiple comparisons. ^*^p < 0.05 and ^**^p < 0.01. (e) Representative F-actin (green) and nuclear (blue) staining of cytokine conditions associated with increased contractility and control group. Scale bar = 2 mm. (f) Total microtissue area as measured by F-Actin fluorescence thresholding. All individual data points represent biological replicates. Conditions are comprised of three biological replicates for four human donors (n = 12 data points per condition). Data represented as mean ± S.E.M. and analyzed by one way ANOVA with Geisser–Greenhouse correction and Dunnett's multiple comparisons. ^*^p < 0.05 and ^***^p < 0.001.

Microtissues of primary human bronchial smooth muscle cells (hBSMCs) from four independent healthy donors were seeded in a 96-well plate with the DEFLCT full plate insert, and tissues were observed in all wells after 24 h [Fig. 2(a) and supplementary material Fig. 4(a)]. Tissues with DEFLCT were manually transferred to a new plate containing starvation media (0.5% FBS) and the panel of inflammatory cytokines (all at 10 ng/ml) [Fig. 2(a)]. Microtissues were cultured until significant tissue shortening was observed in the treatment conditions, which was apparent by day 7. Thus, a 7-day timeframe was used as the experimental end point [Fig. 2(a) and supplementary material Fig. 5). A trained deep learning model was used to measure the day 7 distance between the cantilever tips (T1) and earlier, the unseeded day 0 distance (T0) [Fig. 2(b)]. The deflection was then reported as a percentage of the original tissue length.

We observed that hBSMCs treated with TGF-β1 and IL-13 showed, on average, the greatest decreases in distance between the cantilevers and that these cytokines caused significantly more deflection of the cantilevers compared to the healthy controls [Figs. 2(c) and 2(d)]. Fluorescent staining of the tissues and measurement of the tissue area after fixing and F-actin staining confirmed that the total tissue area decreases in hBSMC microtissues treated with TGF-β1 and IL-13 compared to control tissues, indicating that the tissue tightens with treatment across the four donors [Figs. 2(e) and 2(f)]. These data indicate that we were able to drive microtissues of healthy cells from multiple donors toward the hypercontractile shortened state that is present in asthmatic airways. Furthermore, we have identified TGF-β1 and IL-13 as contractile modulators from among the larger set of asthma associated cytokines.

### TGF-***β***1 and IL-13 cause a shift in the transcriptomic contractility signature

**FIG. 3.**
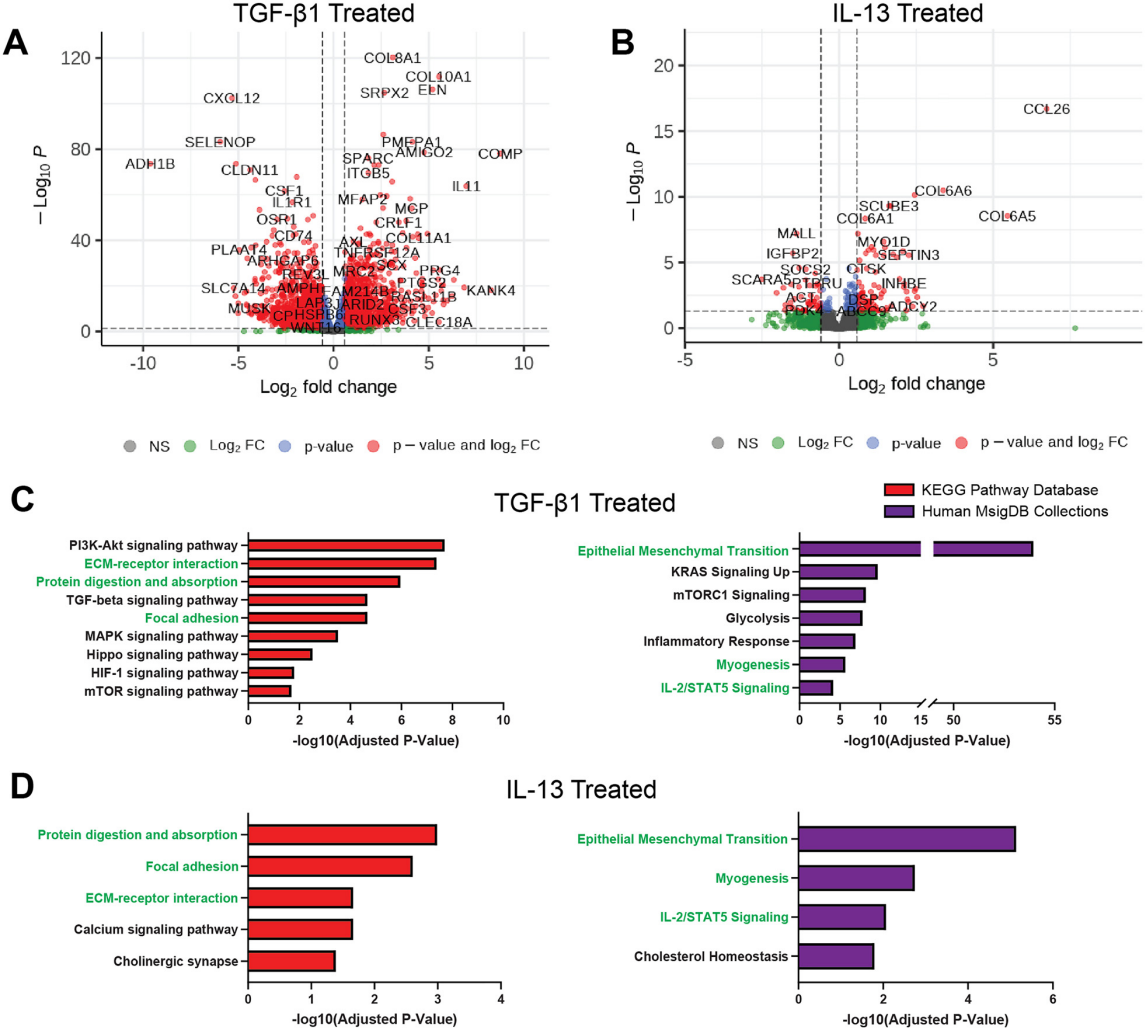
Contractility inducing cytokines independently enrich common pathways associated with asthmatic phenotypes. (a) and (b) Bulk RNA sequencing volcano plots for differentially expressed genes in TGF-β1 (a) and IL-13 (b) treated microtissues. DEGs are defined as having a p value >0.05 and a Log_2_ fold change > 0.585 (FC > 1.5). (c) and (d) Select pathways enriched by DEGs in TGF-β1 (c) and IL-13 (d) treated microtissues associated with an asthmatic SMC phenotype. All shown pathways were curated from the 20 lowest adjusted p-value results (with an absolute minimum adjusted p-value < 0.05) from Enrichr pathway analysis using KEGG Pathway Database (red) and Human MSigDB Collections (purple) for DEGs. Common pathways enriched in both TGF-β1 and IL-13 treated tissues are highlighted in green. A full list of all enriched pathways for each condition and pathway database along with specific associated DEGs can be found in supplementary material Tables S2–S8.

To investigate the transcriptomic changes that underlie the increased contractility and decreased tissue area observed after TGF-β1 and IL-13 treatment, we performed bulk RNA sequencing on microtissues from four independent healthy donors that were treated with 10 ng/ml of TGF-β1 and IL-13. While both TGF-β1 and IL-13 caused significant increases in applied force and decreases in tissue area, they differed drastically in their differentially expressed gene (DEG) signature. TGF-β1 treatment resulted in 3688 DEGs, and IL-13 treatment resulted in 138 DEGs, which had a p-value less than 0.05 and a fold change greater than 1.5 (|Log2FC| > 0.585) [Figs. 3(a) and 3(b), supplementary material Tables S2–S4). DEGs that were upregulated in each condition were input into Enrichr^35^ to obtain the key pathways of interest. Alignment to the KEGG pathway database and the human MSigDB Hallmark collections revealed several overlapping pathways of interest in both TGF-β1 and IL-13 treated microtissues [Figs. 3(c) and 3(d)]. The full list of pathways can be seen in supplementary material Tables S5–S8.

Both treatments resulted in an enrichment of pathways associated with protein digestion and absorption, focal adhesion, and extracellular matrix (ECM)–receptor interaction, which correlates with the observed phenotypic changes in the microtissues by the smooth muscle [Figs. 3(c) and 3(d)]. A closer observation of the top DEGs in both conditions show an upregulation in the expression of matrix proteins, including a variety of collagen subfamily proteins and elastin (ELN), as well as proteins involved in matrix modifications such as matrix metalloprotease 2 (MMP2) and lysyl oxidase (LOX), which can cross-link collagen fibers^36^ and is upregulated in both conditions (supplementary material Fig. 6). The two treatment conditions also show an enrichment in epithelial to mesenchymal transition (EMT), the myogenesis pathway, and IL-2/STAT5 signaling [Figs. 3(c) and 3(d)]. Individually, TGF-β1 treatment shows an enrichment in PI3K-Akt, MAPK, Hippo, HIF-1, mTOR, and KRAS pathways, while IL-13 treated microtissues show a significant enrichment in calcium signaling, cholinergic synapse, and cholesterol homeostasis pathways. Cholesterol homeostasis is also significantly enriched in the TGF-β1 treated microtissue, which was not included in Fig. 3 but can be seen in supplementary material Table S6. Combined with the observed phenotypic changes, these findings show pathways-level changes that might be responsible for the increased contractility. Furthermore, this model can be used for therapeutic drug discovery to identify compounds that reverse the shortened airways.

### Kinase activity robustly modulates airway smooth muscle contractility following TGF-***β***1 signaling

**FIG. 4.**
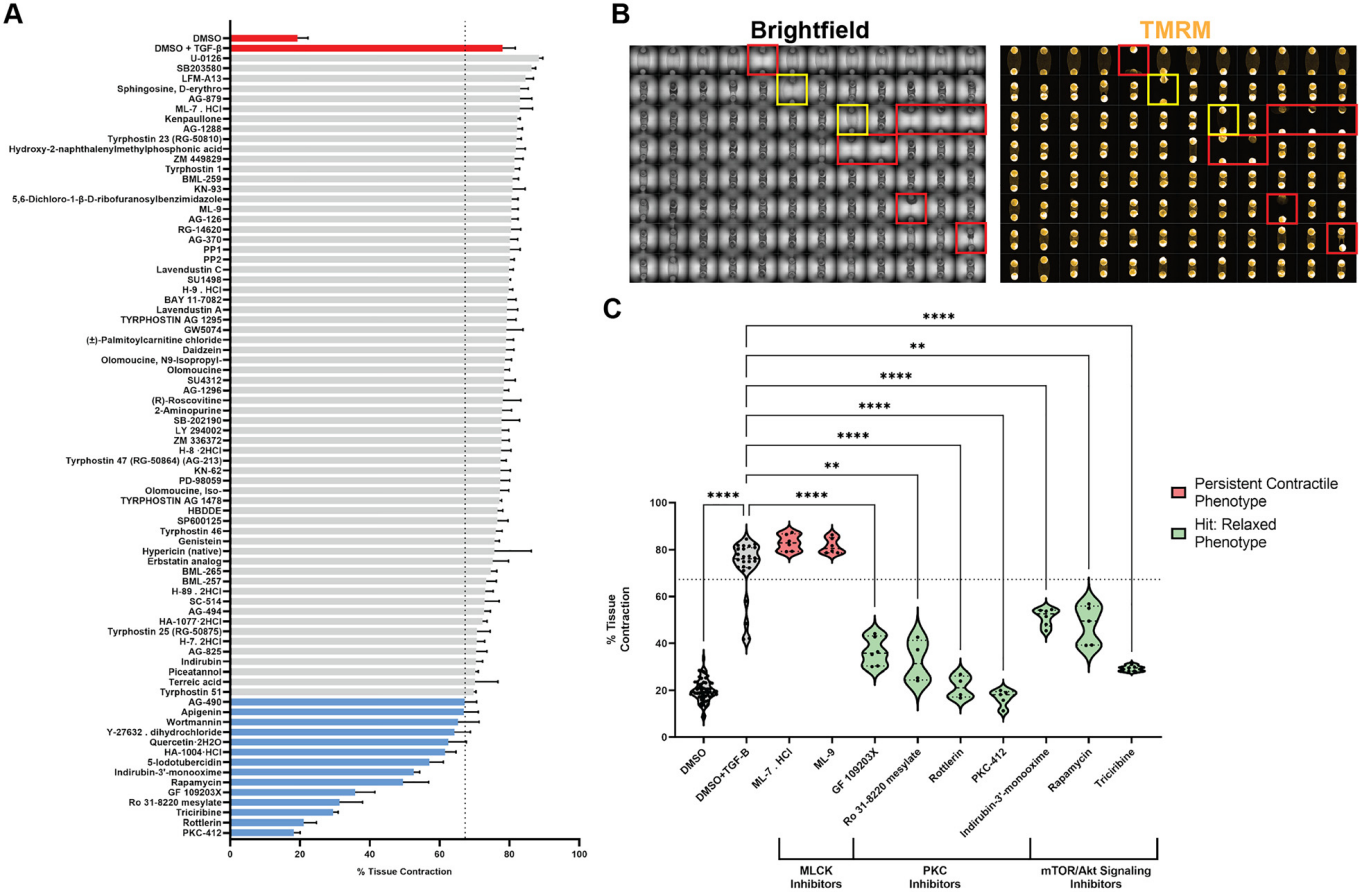
Kinase inhibitor screening demonstrates multiple routes for preventing TGF-β1 induced contractility in BSMC microtissues. (a) Kinase inhibitor library screening for tissue contractility (78 compounds tested) at 1 *μ*M dosing. Bar plot shows robust statistical measurements (median ± median absolute deviation). Hits (blue) are defined by a reduction in contractility greater than three times the median absolute deviation of present contractility for TGF-β1 treated control group (red) represented by dashed axis at 67.29% tissue contraction. Data represents n = 2 biological replicates. (b) Representative full plate images of brightfield and TMRM viability staining (orange) preformed for all screening replicates. Wells shown in red represent exclusions based on damaged/detached tissues by day 7, while wells shown in yellow represent dead tissues assessed via TMRM staining. Well B6 represents the screen's dead control of 1 *μ*M staurosporine. (c) Violin plots for selected kinase inhibitors grouped by inhibitor target. Hits (green) represent the seven least contracted kinase inhibitor conditions, while non-hits (red) represent all MLCK inhibitors within the library. Data were analyzed by one way Brown–Forsythe and Welch ANOVA, with Dunnett's multiple comparisons. ^**^p < 0.01, ^***^p < 0.001, and ^****^p < 0.0001. Data represents n = 2 biological replicates with a minimum of two technical replicates comprising each biological n. Dashed axis annotates tissue contraction < 67.29%.

In addition to gene expression, transient signaling mechanisms, such as kinase and phosphatase activity, also govern airway smooth muscle mechanics.^13–16,37,38^ To explore the role of these acute signals in regulating cytokine induced contractility, we performed a drug screen using 78 known kinase inhibitors on TGF-β1 treated microtissues over a 7-day period at a fixed 1 *μ*M dose. TGF-b1 and kinase inhibitor were simultaneously dosed at days 1 and 4 of culture. A full list of compounds, including their associated protein/pathway targets, is provided in supplementary material Table S9. At day 7 and 14 of these inhibitors were identified as “hits” that significantly reduced tissue contraction after cytokine treatment by more than 3× the median absolute deviation of the TGF-β1 treated control group [Figs. 4(a) and 4(b), supplementary material Fig. 7(a)]. Tissues were assessed visually for damage or detachment following compound treatment, and such wells were excluded from the dataset [Fig. 4(b)]. Additionally, viability for all compounds was assessed via TMRM staining relative to live and dead controls with no significant compound toxicity at the 1 *μ*M dose noted for any hits [Fig. 2(b), supplementary material Figs. 8(a) and 8(b)].

Interestingly, the seven kinase inhibitors that resulted in the least amount of deflection with TGFB1 treatment were either protein kinase C isoforms or elements of the mTOR/Akt signaling cascade, the latter of which coincides with previously noted mTOR pathway enrichment in TGF-β1 treated tissues via gene expression [Fig. 4(c)]. The inhibitor Y-27632, which targets rho-associated protein kinase (ROCK), also qualified as a screen hit, aligning with its well-established link to tissue contraction via inhibition of myosin light chain phosphatase^16,39^ and serving as an additional validation of the DEFLCT model [supplementary material Fig. 9(a)]. Surprisingly, despite its well-established role in inducing cell contraction, neither of the two myosin light chain kinase (MLCK) inhibitors in the library significantly altered the contractile phenotype, suggesting an independent mechanism for cytokine-induced tightening in hBSMCs [Fig. 4(c)]. Taken together, these data demonstrate the screening capabilities of the DEFLCT system and suggest an MLCK-independent mechanism for TGF-β1 induced airway contraction, which can be attenuated through inhibition of PKC or mTOR/Akt signaling.

## DISCUSSION

The narrowing of the airway by hypercontractile smooth muscle cells is a key feature of airway remodeling that distinguishes asthmatic and healthy airways.^1,5,40,41^ To best be able to identify therapies to slow or halt progression of disease or aid in repair, a high-throughput system that can be used in phenotypic screening is necessary. We have developed a high-throughput platform for multiplexed 3D modeling of contractile microtissues and applied this technology to investigate the impact of a panel of six inflammatory cytokines associated with asthma on hBSMC contractility. We found that the TGF-β1 and IL-13 treated microtissues exhibited a significant increase in contractility, shown by an increase in applied force and resultant decrease in distance between the cantilevers, as well as a significant reduction in tissue area surrounding the posts. While all the tested cytokines included in the panel have been linked to modulation of airway smooth muscle contractility,^21,30–34^ they have not previously been compared using a single readout. This approach, therefore, enables a cleaner determination of which individual cytokines are the strongest drivers of contractility and shortening in the airway smooth muscle across multiple donors. This system could be further applied to investigate synergistic effects of combinatorial cytokine treatments; so, although some of the tested cytokines did not produce changes in contractility and remodeling of the tissues in this isolated hBSMC system, they might affect smooth muscle contractility by increasing sensitivity to contractile agonists.^21,32,33^ The suspended microtissue culture enabled by the DEFLCT system also allows for future studies to investigate the effects of these cytokines in co-culture with other cell types, which may reveal additional effects of these cytokines on airway remodeling.

Previous studies of hypercontractile airway behavior have investigated changes in calcium flux or differences after treatment with a contractile agonist (i.e., histamine and acetylcholine), both of which provide information on acute exacerbations, but not baseline changes consistent with airway remodeling.^42,43^ These baseline changes are often investigated with *in vivo* models or *ex vivo* slices, which are not practical for drug discovery biology.^42–44^ The DEFLCT system was able to identify the important cytokine drivers of smooth muscle contractility and simultaneously provide an avenue to induce a disease phenotype for future investigations on therapeutics for repair without the need for tissue explants or expensive and time-consuming *in vivo* studies.^42,44^ This technology could also potentially reduce the number of animals needed for drug discovery. Furthermore, its simple, robust fabrication process and adaptability to existing ANSI/SLAS labware and automation systems enables easy scalability to larger small molecule, siRNA, and CRISPR screening libraries in future studies.

In addition to inducing hypercontractility and microtissue shortening to phenotypically drive the hBSMCs toward an asthmatic airway phenotype, we employed RNAseq to investigate the transcriptomic changes that underly the shift in contractility. Pathways that were uniquely enriched in TGF-β1 treated microtissues included PI3K-Akt,^38^ MAPK,^45^ and mTOR,^46^ which have all been implicated in asthma. IL-13 treated microtissues also showed an enrichment in calcium signaling,^47^ which plays an integral role in smooth muscle contraction. We found several overlapping pathways in the TGF-β1 and IL-13 treated microtissues, which could explain the similarities in phenotypic changes. In both treatment conditions, upregulation in gene expression of ECM components and modifiers, as well as enrichment of pathways involved in focal adhesions and cell-ECM interactions, supports the argument that the smooth muscle cells under the influence of these cytokines would play a hand in remodeling the airway matrix.^36,48^ Previous studies have shown that treatment of BSMCs with serum from asthmatic patients increases the production of ECM proteins, which further supports the link between inflammation and remodeling induced by airway smooth muscle.^49^ EMT has been previously implicated in asthma^50,51^ though not particularly in the context of airway smooth muscle, given its already mesenchymal lineage.^52^ However, upregulation in EMT-associated proteins could potentially signify a transition of the airway smooth muscle cells into a more contractile phenotype and further support a shift toward airway remodeling. Similarly, increases in pathways involved in myogenesis,^53^ cholesterol homeostasis,^54^ and STAT5 signaling^55^ can also be indicative of a shift toward the asthma phenotype, as all three pathways have been shown to play a role in allergic asthma inflammation and smooth muscle cell contractility. Given the link between many of the highlighted pathways with asthma and the changes in the contractile phenotype of the microtissues, we believe our system provides an important high-throughput avenue for investigation of therapeutics for the repair and reversal of airway smooth muscle hypercontractility and remodeling.

Finally, we employed kinase inhibitor screening to explore which mechanisms may be transducing the TGF-β1 signaling into a tightening of airway smooth muscle tissue. We observed clear reductions in contractility among PKC and mTOR/Akt targeted inhibitors, but not in the key contractile protein MLCK. These observations are strengthened by the redundancy of targets within the screening library as multiple inhibitors of the same pathway cause near identical reductions in contractility, which limits the risk of off target effects skewing results. The absence of a relaxed phenotype following MLCK inhibition may suggest that a mechanism independent of calcium signaling is responsible for this cytokine induced hypercontractility.^13,14,47^ Future work will likely focus on clarifying which, if any, of the hit signaling mechanisms overlap with other contractile cytokines such as IL-13. The Global Initiative for Asthma (GINA) treatment strategy guidelines recommend inhaled corticosteroids (ICs) coupled with a long acting β2 adrenergic receptor agonist (LABAs) for mild to moderate asthma symptoms.^56^ More recently, agonists of muscarinic receptors (LAMAs) such as tiotropium bromide (trade name Spiriva) have, despite historically inferior performance to β2 agonists as bronchodilators,^57^ found utility as an add on therapy for severe asthma patients not responsive to IC-LABA treatment.^56,58^ Many of the primary kinase pathway hits for TGF-β1 induced contraction observed in the DEFLCT model (PKC, ROCK, and mTOR/Akt) are modulated by activity from the M2 and M3 muscarinic receptors,^59^ and LAMAs have been shown to reduce inflammation generally *in vivo.*^60^ As such this model may recapitulate inflammation-linked mechanisms of muscarinic activity and enable future studies of novel LAMA efficacy via testing against a patient-derived mechanical phenotypic screen. It should also be noted that use of LAMAs without concurrent administration of ICs was associated with elevated risk of severe exacerbations, limiting their add on use to severe disease cases.^56,60^ It is our hope that complex mechanical models like DEFLCT may aid in the discovery of alternative long-term asthma monotherapies that more directly address the specific pathways underlying hBSMC hypercontractility. It is also worth noting that the use of this technology can extend beyond upper airway respiratory diseases to any organ system where contractile cells play a role in or are affected by disease progression, including diseases involving the cardiovascular system,^61^ fibrosis,^62^ or the musculoskeletal system.^63^

There are technical limitations to the DEFLCT airway model, which will require continued development of the system. The organization of *in vivo* smooth muscle tissue is radial rather than linear,^5^ which is not directly recapitulated in the 3D hBSMC constructs and may limit translatability of exact force readouts. Additionally, this study does not address the role of crosstalk between the smooth muscle and other cells within the niche, such as bronchial epithelial or T-helper cells. The easy transfer of DEFLCT consumables between tissue culture treated assay plates enables future co-culture studies whereby smooth muscle constructs are suspended directly above monolayer cultures of disease-relevant cell types. Finally, although the DEFLCT platform enables larger screening studies on the order of hundreds of well replicates, the use of larger and more diverse small molecule libraries common to pharma pipelines containing thousands or tens of thousands of compounds requires still higher throughput assay plate formats. Development of 3D contractility assays compatible with 384 and 1536 well formats could enable such screening experiments, and MEMS technologies may offer a solution to the form factor challenges inherent to such a small volume.

## CONCLUSIONS

We have developed a high-throughput platform for multiplexed 3D microtissues that can provide quantitative measurements of contractility and be easily integrated with existing high-throughput screening platforms. We have applied this technology to understanding the effects of a panel of inflammatory cytokines on airway smooth muscle contractility and found that TGF-β1 and IL-13 induce a hypercontracted microtissue state compared to control. RNAseq analysis reveals that a majority of the distinct and overlapping enriched pathways across the two treatment conditions can be linked to asthma, smooth muscle contractility, and/or airway remodeling. Integration of complex, high-throughput 3D systems into investigating disease biology can help improve investigation of potential pathways for therapeutic intervention.

## METHODS

### Cell culture

Normal human bronchial smooth muscle cells (hBSMCs) were obtained from Lonza (CC-2576) and cultured using SmGM^TM^-2 BulletKit^TM^ (Lonza CC-3182). All cells were from patients ranging in age from 30 to 65 years old and were sub-cultured according to Lonza's recommended protocol.

### Device fabrication

The cantilever consumables were fabricated in either an 8w strip designed to fit into a microplate's column or a full 96w array. The methods described represent fabrication techniques for both consumable sizes. Cantilever consumables were made by curing liquid silicone rubber (LSR) inside a machined aluminum mold. Equal amounts (1:1 mix ratio, parts A and B) of LSR (NuSil MED-4940) were mixed inside a vacuum planetary mixer (THINKY MIXER ARV-310P) at 1800 rpm for a total approximate duration of 6 min. Once thoroughly mixed and degassed, a pneumatic dispensing gun (Semco^®^ 250-A) was used to inject the LSR into a mold. The uncured LSR was vulcanized at 150 °C for 2 min using a hydraulic lab press equipped with heated platens (Carver Bench Top Auto Press) and then demolded from the tool.

A rigid acrylic backing sheet was attached to the sheet of soft silicone cantilevers to provide a stiff supportive layer facilitating ease of use. A tabletop laser cutter (Glowforge Pro) was used to cut the acrylic backing sheets for both types of cantilever consumables. A double-sided pressure sensitive adhesive (PSA) (3M 96042) was used to attach the silicone cantilevers to the laser cut acrylic backing sheets. The PSA was cut to the appropriate geometry using a craft cutter (Silhouette Cameo 4).

### Mechanical testing and finite elements modeling

Tensile testing samples were prepared as described above in 5 × 24 × 1 mm^3^ rectangular strips. Samples were clamped into an Instron universal testing system (Instron, Norwood, MA) with a 500N load cell equipped. Samples were elongated at a rate of 5 mm/min until the component failed by tearing. Young's modulus and ultimate tensile strain were determined for each curve using Bluehill Universal software (Instron, Norwood MA).

FE model of the hanging posts was created in SimScale CAE software (SimScale GmbH). A second order finite mesh with 24 777 tetrahedral elements and 42 203 nodes was generated through an automatic mesh generator tool that is part of SimScale. Post material was considered to be incompressible, having Young's modulus E = 1.33 MPa, Poisson's ratio ν = 0.499, and density ρ = 1120 kg/m^2^. The posts were treated as cantilevers and an inward force was applied to the caps to induce deflection and simulate tissue contraction. The force was evenly distributed over the surface area of the caps and symmetrically applied to each post to pull them together. Simulated deflection as a function of applied force was computed and plotted.

### Device seeding and cytokine treatment

Cantilever arrays were initially treated with 100% oxygen plasma for 3 min (Nordson Electronic Solutions, Concord, CA) and then immediately submerged and incubated in a 0.15 mg/ml type 1 collagen solution (Advanced Biomatrix 5153) for 1 h to coat the silicone exterior. The 96-well plates (Perkin Elmer 6055302) were pretreated with an anti-adhesion rinse solution (Stem Cell Technologies 07010) for a minimum of 15 min to prevent cell attachment. Cells were then suspended in a 1 mg/ml neutralized type 1 collagen solution then dispensed at a density of 50 000 cells per well into the plate over ice. Cantilever arrays were immediately added to the well, ensuring the ends of each cantilever were fully submerged and incubated at 37 °C for 15 min to allow for gelation. Growth media was then added to the seeded wells, and plates were incubated overnight to allow for initial tissue formation (day 0). All biological replicates for all experiments were generated using new consumables, and pillar arrays were never re-used to lower risk of experiment-to-experiment contamination.

Following 24 h of incubation (day 1), cantilever arrays with seeded microtissues were transferred into a fresh 96-well plate containing hBSMC growth media with reduced serum (0.5% FBS) and the indicated recombinant cytokines at 10 ng/ml (see supplementary material Table S1 for catalog numbers). Microtissues were incubated for 3 days, after which the media and cytokines were replenished (day 4). After an additional 3 days of treatment (day 7), microtissues were used for desired end point assessment.

### Live cell staining via TMRM

Tetramethylrhodamine methyl ester perchlorate (TMRM) stain was obtained from Thermo Fisher (Cat. T668, Thermo Fisher). Tissues were incubated 3 and 7 days in starvation media containing TMRM at a concentration of 25 nM prior to imaging. Tissues were then imaged at 5× and 20× magnification using an Opera Phenix High-Content Imager (Perkin Elmer). Images acquired at 5× magnification were compiled from four fields of view. Images at 20× were acquired across the tissue midline and compiled from six fields of view. All images and cross-sectional reconstructions are represented as maximum intensity projections. Viability was visually assessed relative to a dead control of 1 *μ*M staurosporine (supplementary material Fig. 9).

### Contractility analysis

Prior to seeding, brightfield images of the unseeded cantilever arrays were acquired on day 0 using an Opera Phenix High-Content Imager (Perkin Elmer) to calculate the initial distance between the pillar caps (D0). At day 7 of cytokine treatment, brightfield images of the cantilever arrays with tissues were acquired again to calculate the distance between the pillars after treatment (D7). After the Phenix acquired and exported images to storage servers, an in-house pipeline with the following functions was applied sequentially: background and shading correct (BaSiC),^64^ image stitching, and maximum intensity projection. The final outputs of 2160 × 2160 pixels were used during the evaluation of immunofluorescent staining and cantilever distance measurement. For tissue area segmentation, the image size was reduced to 540 × 540 pixels by 4 × 4 binning. The pipeline recorded pixel and voxel size (*μ*m) from metadata for the downstream calculation. We performed data annotation in Napari^65^ by using a random-forest-based segmentation plugin named napari-accelerated-pixel-and-object-classification (APOC),^66^ paired with our customized image sampler and label cleaner, to create masks for both post and microtissue labeling.

Our method of analysis integrates Medical Open Network for Artificial Intelligence (MONAI)^67^ and PyTorch^68^ for neural network training, utilizing the MONAI transform function to perform on-the-fly data augmentation in the training process. Selected transform functions include spatial crops (RandSpatialCropSamplesd), random flip (RandFlipd), scale and shift intensity (RandScaleIntensityd and RandShiftIntensityd), and 90° rotate (RandRotate90d). Random probabilities for each transform function are assigned individually. We selected a cropping size of 192 × 192 pixels, sampling 40 times per image. In total, 173 datasets were used during model training.

The neural network architecture used in our method is two residual units, five layers of Res-UNet.^69^ The loss function is the sum of Dice loss per batch in a batch size of 32. We performed our model training using an Nvidia Tesla V100-SXM2 GPU with 32 GB memory (Nvidia, Santa Clara, California). The training process ran within 500 epochs and was optimized by Adam optimizer,^70^ with an initial learning rate of 0.001. For validation, 20% of training datasets were randomly chosen, and our workflow selected the model with the highest Dice metrics for the following image segmentation.

Once the U-Net model predicted the regions of posts, we used rule-based methods, assisted by human inspection, to ensure the quality of segmentation results. Later, our approach generated the distance map with Euclidean distance transformation in SciPy,^71^ detecting the minimum distance between two posts. The results are reported in both pixel and *μ*m. Displacement was then converted to % tissue contraction by comparing initial and final displacement values.

### Bradykinin treatment

Bradykinin acetate salt was obtained from Sigma (Millipore Sigma, B3259) and diluted to a 23.58 mM stock solution. Tissues were imaged for baseline tightening as described above on day 3 of culture. Then, Bradykinin stock was further diluted in starvation media and added to microtissues for a final dose of 100 *μ*M. Treated tissues were allowed to incubate for 12 min and then imaged for their contracted state. The resulting acquisitions were then processed using the analysis method described above.

### Actin staining and analysis

Microtissues were transferred to wells with 4% PFA in PBS (diluted from 32% PFA solution, Electron Microscopy Sciences 15714-S) for 15 min. Tissues were then washed three times with PBS and blocked with 1% BSA (Millipore Sigma A2153) for 20 min, after which they were transferred to a staining solution containing Alexa Fluor 488 phalloidin (Thermo Fisher A12379) and Hoechst (Thermo Fisher 62249) and incubated overnight at room temperature on a shaker. Tissues were then washed three times with PBS and imaged at 5× magnification using an Opera Phenix High-Content Imager (Perkin Elmer) with four fields of view to capture the full microtissues, which were stitched before calculating the total tissue area.

To calculate the area of the stained tissue, we employed a similar workflow to train the prediction model, but with some modifications. Changes included the following: (1) input images used for Res-UNet training were cropped from images with original pixel size and cropped into size of 384 × 384 pixels. (2) We scaled the intensity with a fixed range (ScaleIntensityRanged) calculated from our datasets. (3) The training was run with a batch size of six within 600 epochs. With model predictions, our method applied region property measurement from scikit-image^72^ and returned area in pixel2 and mm^2^.

### RNA sequencing

RNA was isolated from hBSMC microtissues that were either untreated or treated with TGF-β1 and IL-13 for 7 days prior to RNA isolation using the RNeasy Mini Kit (Qiagen 74106). The RNA yield was measured using Agilent TapeStation (4200 TapeStation System) at 260 nm. A quantity of 200 ng of total RNA was used to make RNA sequencing libraries using the TruSeq Stranded mRNA Prep Kit (Illumina 20020595). Libraries were run on a 51-cycle NovaSeq (SP flowcell, two lanes) sequencing run and yielded an average sequencing depth of 8 × 10^6^ mapped reads per sample. Reads were generated from a NovaSeq instrument with bcl2fastq from Illumina, v 2.20.0.422–2, and were aligned to a genome and annotation from Ensembl, assembly GRCh38.p13, release version 104.^73^ Gene counts were assessed with RSEM v1.3.^74^ Differential gene expression was assessed with DESeq2 v.1.28.1, in R version 4.2.0.^75^ All conditions consist of RNA from four independent donors from n = 2–3 biological replicates. DEGs that were upregulated in each condition were input into Enrichr^35^ to obtain the key pathways of interest.

### Kinase screening

Kinase inhibitor libraries were purchased from Enzo Life Sciences (Screen-Well BML-2832-0100) and diluted to a stock concentration of 1 mM in DMSO. A full table of library contents, structures, and targets is included in supplementary material Table S9. Stocks were then further diluted in starvation cell culture media and then immediately transferred to 96w plates containing media and TGF-β1 as described above such that the final compound concentration was 1 *μ*M. Seeded DEFLCT consumables were then transferred into these pretreated well plates and cultured for 3 days. Compounds were initially added on day 1 of culture and then again on day 4 via the same process. All compound transfers were performed on a Bravo Automated Liquid Handling Platform (Agilent) inside a sterile enclosure. Tissues were visually assessed for damage or detachment as well as viability on all technical and biological replicates prior to inclusion in data cohorts. The results were filtered as “hits” based on a contractility reduction > 3× the median absolute deviation of the TGF-β1 treated control group [supplementary material Fig. 7(a)].

### Statistical analysis

All experiments were performed using cells from four different donors obtained from Lonza and n biological replicates as indicated in each figure where appropriate. Bar graphs are represented as mean ± SEM, excepting kinase screen results, which is represented by median ± M.A.D. Statistical analyses were performed using GraphPad Prism9, the threshold for significance level is set at P < 0.05. Screen hits were determined by a reduction in contractility greater than 3× M.A.D. of TGF-β1 treated controls. A detailed information on analyses is indicated in the figure legends.

## SUPPLEMENTARY MATERIALS

See the supplementary material for gene expression data tables (supplementary material Tables S2–S8) generated from RNAseq analysis of TGF-β1 and IL-13 treated microtissues.

## Data Availability

The data that support the findings of this study are available within the article and its supplementary material. The data that support the findings of this study are openly available in NCBI GEO at Accession No. GSE220972, Ref. 76.

## References

[1] I. Bara , A. Ozier , J.-M. Tunon de Lara , R. Marthan , and P. Berger , Eur. Respir. J. 36, 1174 (2010). 10.1183/09031936.00019810 21037369

[2] T. Doherty and D. Broide , Curr. Opin. Immunol. 19, 676 (2007). 10.1016/j.coi.2007.07.017 17720466

[3] R. Halwani , S. Al-Muhsen , H. Al-Jahdali , and Q. Hamid , Am. J. Respir. Cell Mol. Biol. 44, 127 (2011). 10.1165/rcmb.2010-0027TR 20525803

[4] S. Zuyderduyn , M. B. Sukkar , A. Fust , S. Dhaliwal , and J. K. Burgess , Eur. Respir. J. 32, 265 (2008). 10.1183/09031936.00051407 18669785

[5] D. C. Doeing and J. Solway , J. Appl. Physiol. 114, 834 (2013). 10.1152/japplphysiol.00950.2012 23305987 PMC3633438

[6] S. S. An , T. R. Bai , J. H. T. Bates , J. L. Black , R. H. Brown , V. Brusasco , P. Chitano , L. Deng , M. Dowell , D. H. Eidelman , B. Fabry , N. J. Fairbank , L. E. Ford , J. J. Fredberg , W. T. Gerthoffer , S. H. Gilbert , R. Gosens , S. J. Gunst , A. J. Halayko , R. H. Ingram , C. G. Irvin , A. L. James , L. J. Janssen , G. G. King , D. A. Knight , A. M. Lauzon , O. J. Lakser , M. S. Ludwig , K. R. Lutchen , G. N. Maksym , J. G. Martin , T. Mauad , B. E. McParland , S. M. Mijailovich , H. W. Mitchell , R. W. Mitchell , W. Mitzner , T. M. Murphy , P. D. Paré , R. Pellegrino , M. J. Sanderson , R. R. Schellenberg , C. Y. Seow , P. S. P. Silveira , P. G. Smith , J. Solway , N. L. Stephens , P. J. Sterk , A. G. Stewart , D. D. Tang , R. S. Tepper , T. Tran , and L. Wang , Eur. Respir. J. 29, 834 (2007). 10.1183/09031936.00112606 17470619 PMC2527453

[7] D. B. Wright , T. Trian , S. Siddiqui , C. D. Pascoe , J. R. Johnson , B. G. J. Dekkers , S. Dakshinamurti , R. Bagchi , J. K. Burgess , V. Kanabar , and O. O. Ojo , Pulm. Pharmacol. Ther. 26, 42 (2013). 10.1016/j.pupt.2012.08.005 22939888

[8] N. T. Mendonça , J. Kenyon , A. S. LaPrad , S. N. Syeda , G. T. O'Connor , and K. R. Lutchen , Respir. Res. 12, 96 (2011). 10.1186/1465-9921-12-96 21762517 PMC3143926

[9] E. Wang , M. E. Wechsler , T. N. Tran , L. G. Heaney , R. C. Jones , A. N. Menzies-Gow , J. Busby , D. J. Jackson , P. E. Pfeffer , C. K. Rhee , Y. S. Cho , G. W. Canonica , E. Heffler , P. G. Gibson , M. Hew , M. Peters , E. S. Harvey , M. Alacqua , J. Zangrilli , L. Bulathsinhala , V. A. Carter , I. Chaudhry , N. Eleangovan , N. Hosseini , R. B. Murray , and D. B. Price , Chest 157, 790 (2020). 10.1016/j.chest.2019.10.053 31785254

[10] E. Israel and H. K. Reddel , New England J. Med. 377, 965 (2017). 10.1056/NEJMra1608969 28877019

[11] T. D. Pollard , R. R. Weihing , and M. R. Adelman , CRC Crit. Rev. Biochem. 2(1), 1–65 (1974). 10.3109/10409237409105443 4273099

[12] R. C. Webb , Adv. Physiol. Educ. 27, 201 (2003). 10.1152/advances.2003.27.4.201 14627618

[13] F. Hong , B. D. Haldeman , D. Jackson , M. Carter , J. E. Baker , and C. R. Cremo , Arch. Biochem. Biophys. 510, 135 (2011). 10.1016/j.abb.2011.04.018 21565153 PMC3382066

[14] H. Kishi , L.-H. Ye , A. Nakamura , T. Okagaki , A. Iwata , T. Tanaka , and K. Kohama , *Mechanisms of Work Production and Work Absorption in Muscle* ( Plenum Press, New York,1998), Vol. 229.

[15] H. Sakai , Y. Chiba , and M. Misawa , Pulm. Pharmacol. Ther. 20, 734 (2007). 10.1016/j.pupt.2006.08.011 17071121

[16] M. D. Álvarez-Santos , M. Álvarez-González , S. Estrada-Soto , and B. Bazán-Perkins , Front. Physiol. 11, 701 (2020). 10.3389/fphys.2020.00701 32676037 PMC7333668

[17] I. Pushkarsky , P. Tseng , D. Black , B. France , L. Warfe , C. J. Koziol-White , W. F. Jester , R. K. Trinh , J. Lin , P. O. Scumpia , S. L. Morrison , R. A. Panettieri , R. Damoiseaux , and D. di Carlo , Nat. Biomed. Eng. 2, 124 (2018). 10.1038/s41551-018-0193-2 31015629 PMC6619436

[18] S. An , A. Levchenko , K. Ahn , P. Kogut , C. Koziel-White , R. Wang , D. Lee , B. Camoretti-Mercado , R. Panettieri , and J. Solway , *B29. THE Lung on the Border between Order and CHAOS* ( American Thoracic Society, 2012), p. A2694.

[19] D. Wright , P. Sharma , M.-H. Ryu , P.-A. Rissé , M. Ngo , H. Maarsingh , C. Koziol-White , A. Jha , A. J. Halayko , and A. R. West , Pulm. Pharmacol. Ther. 26, 24 (2013). 10.1016/j.pupt.2012.08.006 22967819

[20] C. C. Ceresa , A. J. Knox , and S. R. Johnson , Am. J. Physiol.-Lung Cell. Mol. Physiol. 296, L1059 (2009). 10.1152/ajplung.90445.2008 19346431

[21] M. Kudo , A. C. Melton , C. Chen , M. B. Engler , K. E. Huang , X. Ren , Y. Wang , X. Bernstein , J. T. Li , K. Atabai , X. Huang , and D. Sheppard , Nat. Med. 18, 547 (2012). 10.1038/nm.2684 22388091 PMC3321096

[22] J. E. Bourke , X. Li , S. R. Foster , E. Wee , H. Dagher , J. Ziogas , T. Harris , J. V. Bonacci , and A. G. Stewart , Eur. Respir. J. 37, 173 (2011). 10.1183/09031936.00008109 20595143

[23] F. Lin , H. Zhang , J. Huang , and C. Xiong , Ann. Biomed. Eng. 46, 2000 (2018). 10.1007/s10439-018-2098-3 30051243

[24] C. T. D. Dickman , V. Russo , K. Thain , S. Pan , S. T. Beyer , K. Walus , S. Getsios , T. Mohamed , and S. J. Wadsworth , FASEB J. 34, 1652 (2020). 10.1096/fj.201901063RR 31914670

[25] F. Pampaloni , E. G. Reynaud , and E. H. K. Stelzer , Nat. Rev. Mol. Cell Biol. 8, 839 (2007). 10.1038/nrm2236 17684528

[26] W. R. Legant , A. Pathak , M. T. Yang , V. S. Deshpande , R. M. McMeeking , and C. S. Chen , Proc. Natl. Acad. Sci. U. S. A. 106, 10097 (2009). 10.1073/pnas.0900174106 19541627 PMC2700905

[27] K. Ronaldson-Bouchard , K. Yeager , D. Teles , T. Chen , S. Ma , L. Song , K. Morikawa , H. M. Wobma , A. Vasciaveo , E. C. Ruiz , M. Yazawa , and G. Vunjak-Novakovic , Nat. Protoc. 14, 2781 (2019). 10.1038/s41596-019-0189-8 31492957 PMC7195192

[28] A. S. T. Smith , S. M. Luttrell , J.-B. Dupont , K. Gray , D. Lih , J. W. Fleming , N. J. Cunningham , S. Jepson , J. Hesson , J. Mathieu , L. Maves , B. J. Berry , E. C. Fisher , N. J. Sniadecki , N. A. Geisse , and D. L. Mack , J. Tissue Eng. 13, 20417314221122130 (2022). 10.1177/20417314221122127 36082311 PMC9445471

[29] K. Ronaldson-Bouchard , D. Teles , K. Yeager , D. N. Tavakol , Y. Zhao , A. Chramiec , S. Tagore , M. Summers , S. Stylianos , M. Tamargo , B. M. Lee , S. P. Halligan , E. H. Abaci , Z. Guo , J. Jacków , A. Pappalardo , J. Shih , R. K. Soni , S. Sonar , C. German , A. M. Christiano , A. Califano , K. K. Hirschi , C. S. Chen , A. Przekwas , and G. Vunjak-Novakovic , Nat. Biomed. Eng. 6, 351 (2022). 10.1038/s41551-022-00882-6 35478225 PMC9250010

[30] C. A. Ojiaku , G. Cao , W. Zhu , E. J. Yoo , M. Shumyatcher , B. E. Himes , S. S. An , and R. A. Panettieri , Am. J. Respir. Cell Mol. Biol. 58, 575 (2017). 10.1165/rcmb.2017-0247OC PMC5946330 28984468

[31] M. L. Manson , J. Säfholm , A. James , A.-K. Johnsson , P. Bergman , M. Al-Ameri , A.-C. Orre , C. Kärrman-Mårdh , S.-E. Dahlén , and M. Adner , J. Allergy Clin. Immunol. 145, 808 (2020). 10.1016/j.jaci.2019.10.037 31805312

[32] H. Chen , O. Tliba , C. R. van Besien , R. A. Panettieri , and Y. Amrani , J. Appl. Physiol. 95, 864 (2003). 10.1152/japplphysiol.00140.2003 12730147

[33] J. L. Pype , H. Xu , M. Schuermans , L. J. Dupont , W. I. M. Wuyts , J. C. W. Mak , P. J. Barnes , M. G. Demedts , and G. M. Verleden , Am. J. Respir. Crit. Care Med. 163, 1010 (2001). 10.1164/ajrccm.163.4.9911091 11282781

[34] M. E. Kuruvilla , F. E.-H. Lee , and G. B. Lee , Clin. Rev. Allergy Immunol. 56, 219 (2019). 10.1007/s12016-018-8712-1 30206782 PMC6411459

[35] Z. Xie , A. Bailey , M. v Kuleshov , D. J. B. Clarke , J. E. Evangelista , S. L. Jenkins , A. Lachmann , M. L. Wojciechowicz , E. Kropiwnicki , K. M. Jagodnik , M. Jeon , and A. Ma'ayan , Curr. Protoc. 1, e90 (2021). 10.1002/cpz1.90 33780170 PMC8152575

[36] J. Ramis , R. Middlewick , F. Pappalardo , J. T. Cairns , I. D. Stewart , A. E. John , S.-U.-N. Naveed , R. Krishnan , S. Miller , D. E. Shaw , C. E. Brightling , L. Buttery , F. Rose , G. Jenkins , S. R. Johnson , and A. L. Tatler , Eur. Respir. J. 60, 2004361 (2022). 10.1183/13993003.04361-2020 34996828 PMC9260127

[37] A. M. Trzcińska-Daneluti , L. Nguyen , C. Jiang , C. Fladd , D. Uehling , M. Prakesch , R. Al-awar , and D. Rotin , Mol. Cell. Proteomics 11, 745 (2012). 10.1074/mcp.M111.016626 22700489 PMC3434788

[38] E. J. Yoo , C. A. Ojiaku , K. Sunder , and R. A. Panettieri , Am. J. Respir. Cell Mol. Biol. 56, 700 (2016). 10.1165/rcmb.2016-0308TR PMC5516292 27977296

[39] H. Sakai , W. Suto , Y. Kai , and Y. Chiba , J. Smooth Muscle Res. 53, 37 (2017). 10.1540/jsmr.53.37 28484126 PMC5411784

[40] C. Y. Seow , R. R. Schellenberg , and P. D. Paré , Am. J. Respir. Crit. Care Med. 158, S179 (1998). 10.1164/ajrccm.158.supplement_2.13tac160 9817743

[41] A. Ozier , B. Allard , I. Bara , P.-O. Girodet , T. Trian , R. Marthan , and P. Berger , J. Allergy 2011, 742710. 10.1155/2011/742710 PMC3246780 22220184

[42] J. Chen , M. Miller , H. Unno , P. Rosenthal , M. J. Sanderson , and D. H. Broide , J. Allergy Clin. Immunol. 142, 207 (2018). 10.1016/j.jaci.2017.08.015 28889952 PMC5842097

[43] A. K. Pham , M. Miller , P. Rosenthal , S. Das , N. Weng , S. Jang , R. C. Kurten , J. Badrani , T. A. Doherty , B. Oliver , and D. H. Broide , JCI Insight 6, e136911 (2021). 10.1172/jci.insight.136911 33661765 PMC8119187

[44] A. Brown , J. Danielsson , E. A. Townsend , Y. Zhang , J. F. Perez-Zoghbi , C. W. Emala , and G. Gallos , Am. J. Physiol.-Lung Cell. Mol. Physiol. 310, L747 (2016). 10.1152/ajplung.00215.2015 26773068 PMC4836109

[45] W. T. Gerthoffer and C. A. Singer , Respir. Physiol. Neurobiol. 137, 237 (2003). 10.1016/S1569-9048(03)00150-2 14516729

[46] Y. Zhang , Y. Jing , J. Qiao , B. Luan , X. Wang , L. Wang , and Z. Song , Sci. Rep. 7, 4532 (2017). 10.1038/s41598-017-04826-y 28674387 PMC5495772

[47] J. A. Jude , M. E. Wylam , T. F. Walseth , and M. S. Kannan , Proc. Am. Thorac. Soc. 5, 15 (2008). 10.1513/pats.200704-047VS 18094080 PMC2645299

[48] E. R. Vogel , R. D. Britt , A. Faksh , I. Kuipers , H. Pandya , Y. S. Prakash , R. J. Martin , and C. M. Pabelick , Pediatr. Res. 81, 376 (2017). 10.1038/pr.2016.218 27925619 PMC5309184

[49] P. R. A. Johnson , J. L. Black , S. Carlin , Q. I. Ge , and P. Anne Underwood , Am. J. Respir. Crit. Care Med. 162, 2145 (2000). 10.1164/ajrccm.162.6.9909111 11112129

[50] T.-L. Hackett , S. M. Warner , D. Stefanowicz , F. Shaheen , D. v Pechkovsky , L. A. Murray , R. Argentieri , A. Kicic , S. M. Stick , T. R. Bai , and D. A. Knight , Am. J. Respir. Crit. Care Med. 180, 122 (2009). 10.1164/rccm.200811-1730OC 19406982

[51] Z.-C. Yang , Z.-H. Qu , M.-J. Yi , Y.-C. Shan , N. Ran , L. Xu , and X.-J. Liu , J. Cell Physiol. 234, 8804 (2019). 10.1002/jcp.27540 30362537

[52] B. Camoretti-Mercado , Transl. Res. 154, 165 (2009). 10.1016/j.trsl.2009.06.008 19766960 PMC2764304

[53] Y.-C. Chen , Y.-H. Tsai , C.-C. Wang , S.-F. Liu , T.-W. Chen , W.-F. Fang , C.-P. Lee , P.-Y. Hsu , T.-Y. Chao , C.-C. Wu , Y.-F. Wei , H.-C. Chang , C.-C. Tsen , Y.-P. Chang , M.-C. Lin , C.-J. Yu , H.-C. Wang , C.-H. Chiang , D.-W. Perng , S.-L. Cheng , J.-Y. Hsu , W.-H. Hsu , T.-R. Hsiue , H.-I. Lin , C.-Y. Wang , Y.-C. Chang , C.-M. Chen , C.-S. Lin , L. Chen , I.-W. Chong , and T.C.T.C. of R.D. (TCORE) Group. Sci. Rep. 11, 5022 (2021). 10.1038/s41598-021-83185-1 33658578 PMC7930096

[54] M. Smet , L. van Hoecke , A. de Beuckelaer , S. vander Beken , T. Naessens , K. Vergote , M. Willart , B. N. Lambrecht , J.-Å. Gustafsson , K. R. Steffensen , and J. Grooten , Immun., Inflammation Dis. 4, 350 (2016). 10.1002/iid3.118 PMC5004289 27621817

[55] J. M. Knight , P. Mandal , P. Morlacchi , G. Mak , E. Li , M. Madison , C. Landers , B. Saxton , E. Felix , B. Gilbert , J. Sederstrom , A. Varadhachary , M. M. Singh , D. Chatterjee , D. B. Corry , and J. S. McMurray , J. Biol. Chem. 293, 10026 (2018). 10.1074/jbc.RA117.000567 29739850 PMC6028980

[56] GINA Report, *Global Strategy for Asthma Management and Prevention* ( GINA, 2022).

[57] P. J. Barnes , Proc. Am. Thorac. Soc. 1, 345 (2004). 10.1513/pats.200409-045MS 16113456

[58] S. Muiser , R. Gosens , M. van den Berge , and H. A. M. Kerstjens , Ann. Allergy, Asthma Immunol. 128(4), 352 (2022). 35074516 10.1016/j.anai.2021.12.020

[59] R. Gosens , J. Zaagsma , H. Meurs , and A. J. Halayko , Respir. Res. 7(1), 73 (2006). 10.1186/1465-9921-7-73 16684353 PMC1479816

[60] L. E. M. Kistemaker , I. S. T. Bos , M. H. Menzen , H. Maarsingh , H. Meurs , and R. Gosens , Respir. Res. 17(1), 13 (2016). 10.1186/s12931-016-0327-6 26846267 PMC4743207

[61] W. Dou , M. Malhi , Q. Zhao , L. Wang , Z. Huang , J. Law , N. Liu , C. A. Simmons , J. T. Maynes , and Y. Sun , Microsyst. Nanoeng. 8, 26 (2022). 10.1038/s41378-021-00344-0 35299653 PMC8882466

[62] M. Asmani , S. Velumani , Y. Li , N. Wawrzyniak , I. Hsia , Z. Chen , B. Hinz , and R. Zhao , Nat. Commun. 9, 2066 (2018). 10.1038/s41467-018-04336-z 29802256 PMC5970268

[63] L. A. Moyle , E. Jacques , and P. M. Gilbert , Curr. Opin. Biomed. Eng. 16, 9 (2020). 10.1016/j.cobme.2020.05.006

[64] T. Peng , K. Thorn , T. Schroeder , L. Wang , F. J. Theis , C. Marr , and N. Navab , Nat. Commun. 8, 14836 (2017). 10.1038/ncomms14836 28594001 PMC5472168

[65] N. Sofroniew , T. Lambert , K. Evans , J. Nunez-Iglesias , G. Bokota , P. Winston , G. Peña-Castellanos , K. Yamauchi , M. Bussonnier , D. Doncila Pop , A. Can Solak , Z. Liu , P. Wadhwa , A. Burt , G. Buckley , A. Sweet , L. Migas , V. Hilsenstein , L. Gaifas , J. Bragantini , J. Rodríguez-Guerra , H. Muñoz , J. Freeman , P. Boone , A. Lowe , C. Gohlke , L. Royer , A. Pierré , H. Har-Gil , and A. McGovern (2022) “napari: a multi-dimensional image viewer for Python.” 10.5281/zenodo.3555620

[66] R. Haase , D. D. Pop , and L. Žigutytė (2022). “Haesleinhuepf/napari-accelerated-pixel-and-object-classification: 0.8.2,” 10.5281/zenodo.6914717

[67] MONAI Consortium. “MONAI: Medical Open Network for AI.” 10.5281/zenodo.6903385 (2022).

[68] A. Paszke , S. Gross , F. Massa , A. Lerer , J. Bradbury , G. Chanan , T. Killeen , Z. Lin , N. Gimelshein , L. Antiga , A. Desmaison , A. Kopf , E. Yang , Z. DeVito , M. Raison , A. Tejani , S. Chilamkurthy , B. Steiner , L. Fang , J. Bai , and S. Chintala , in *Advances in Neural Information Processing Systems*, edited by H. Wallach , H. Larochelle , A. Beygelzimer , F. d'Alché-Buc , E. Fox , and R. Garnett ( Curran Associates, Inc., 2019), Vol. 32, pp. 8024–8035.

[69] E. Kerfoot , J. Clough , I. Oksuz , J. Lee , A. P. King , and J. A. Schnabel , in *Statistical Atlases and Computational Models of the Heart. Atrial Segmentation and LV Quantification Challenges*, edited by M. Pop , M. Sermesant , J. Zhao , S. Li , K. McLeod , A. Young , K. Rhode , and T. Mansi ( Springer International Publishing, Cham, 2019), pp. 371–380.

[70] D. P. Kingma and J. Ba , “Adam: A method for stochastic optimization,” arXiv:1412.6980 (2017).

[71] P. Virtanen , R. Gommers , T. E. Oliphant , M. Haberland , T. Reddy , D. Cournapeau , E. Burovski , P. Peterson , W. Weckesser , J. Bright , S. J. van der Walt , M. Brett , J. Wilson , K. J. Millman , N. Mayorov , A. R. J. Nelson , E. Jones , R. Kern , E. Larson , C. J. Carey , İ. Polat , Y. Feng , E. W. Moore , J. VanderPlas , D. Laxalde , J. Perktold , R. Cimrman , I. Henriksen , E. A. Quintero , C. R. Harris , A. M. Archibald , A. H. Ribeiro , F. Pedregosa , P. van Mulbregt , A. Vijaykumar , A. pietro Bardelli , A. Rothberg , A. Hilboll , A. Kloeckner , A. Scopatz , A. Lee , A. Rokem , C. N. Woods , C. Fulton , C. Masson , C. Häggström , C. Fitzgerald , D. A. Nicholson , D. R. Hagen , D. V. Pasechnik , E. Olivetti , E. Martin , E. Wieser , F. Silva , F. Lenders , F. Wilhelm , G. Young , G. A. Price , G.-L. Ingold , G. E. Allen , G. R. Lee , H. Audren , I. Probst , J. P. Dietrich , J. Silterra , J. T. Webber , J. Slavič , J. Nothman , J. Buchner , J. Kulick , J. L. Schönberger , J. V. de Miranda Cardoso , J. Reimer , J. Harrington , J. L. C. Rodríguez , J. Nunez-Iglesias , J. Kuczynski , K. Tritz , M. Thoma , M. Newville , M. Kümmerer , M. Bolingbroke , M. Tartre , M. Pak , N. J. Smith , N. Nowaczyk , N. Shebanov , O. Pavlyk , P. A. Brodtkorb , P. Lee , R. T. McGibbon , R. Feldbauer , S. Lewis , S. Tygier , S. Sievert , S. Vigna , S. Peterson , S. More , T. Pudlik , T. Oshima , T. J. Pingel , T. P. Robitaille , T. Spura , T. R. Jones , T. Cera , T. Leslie , T. Zito , T. Krauss , U. Upadhyay , Y. O. Halchenko , Y. Vázquez-Baeza , and SciPy 1.0 Contributors, Nat. Methods 17, 261 (2020). 10.1038/s41592-019-0686-2 32015543 PMC7056644

[72] S. van der Walt , J. L. Schönberger , J. Nunez-Iglesias , F. Boulogne , J. D. Warner , N. Yager , E. Gouillart , and T. Yu , PeerJ 2, e453 (2014). 10.7717/peerj.453 25024921 PMC4081273

[73] A. Dobin , C. A. Davis , F. Schlesinger , J. Drenkow , C. Zaleski , S. Jha , P. Batut , M. Chaisson , and T. R. Gingeras , Bioinformatics 29, 15 (2013). 10.1093/bioinformatics/bts635 23104886 PMC3530905

[74] B. Li and C. N. Dewey , BMC Bioinf. 12, 323 (2011). 10.1186/1471-2105-12-323 PMC3163565 21816040

[75] M. I. Love , W. Huber , and S. Anders , Genome Biol. 15, 550 (2014). 10.1186/s13059-014-0550-8 25516281 PMC4302049

[76] P. Beri , C. Plunkett , J. Barbara , C. Shih , S. W. Barnes , O. Ross , P. Choconta , T. Trinh , B. Litvin , J. Walker , M. Qiu , S. Hammack , and E. Toyama , “ Investigating the effects of inflammatory cytokines associated with asthma on airway smooth muscle cell contractility,” Accession No. GSE220972 ( NCBI GEO, 2022).

